# Unpacking the Relationship between Parental Migration and Child well-Being: Evidence from Moldova and Georgia

**DOI:** 10.1007/s12187-017-9461-z

**Published:** 2017-03-25

**Authors:** Franziska Gassmann, Melissa Siegel, Michaella Vanore, Jennifer Waidler

**Affiliations:** Maastricht Graduate School of Governance/UNU-MERIT, Maastricht University, Boschstraat 24, 6211AX, Maastricht, The Netherlands

**Keywords:** Migration, Children, Multi-dimensional poverty, Moldova, Georgia, F22, I32, J14, J61

## Abstract

Using household survey data collected between September 2011 and December 2012 from Moldova and Georgia, this paper measures and compares the multidimensional well-being of children with and without parents abroad. While a growing body of literature has addressed the effects of migration for children ‘left behind’, relatively few studies have empirically analysed if and to what extent migration implies different well-being outcomes for children, and fewer still have conducted comparisons across countries. To compare the outcomes of children in current- and non-migrant households, this paper defines a multidimensional well-being index comprised of six dimensions of wellness: education, physical health, housing conditions, protection, communication access, and emotional health. This paper challenges conventional wisdom that parental migration is harmful for child well-being: while in Moldova migration does not appear to correspond to any positive or negative well-being outcomes, in Georgia migration was linked to higher probabilities of children attaining well-being in the domains of communication access, housing, and combined well-being index. The different relationship between migration and child well-being in Moldova and Georgia likely reflects different migration trajectories, mobility patterns, and levels of maturity of each migration stream.

## Introduction

In many societies experiencing large-scale mobility transitions, migration is often framed in public discourse as either a blessing or a curse for those households and family members “left behind” by migrating kin in the home country. This is true of both Moldova and Georgia, two post-Soviet countries that have experienced the emigration of large shares of their total populations (World Bank 2015).^1^ Such large-scale emigration has inspired growing concern about the potential costs and benefits of migration, particularly for those children who are “left behind” by their migrant parents.

Migration and its consequences are difficult to quantify. Remittances are one of the best-explored outcomes of migration given the substantial financial flows they can represent: in Moldova, remittances accounted for over 24% of GDP in 2014 and in Georgia, 12% (World Bank 2015). Such remittance flows can play a key role in protecting recipient households from economic shocks and income vulnerability, yet it is unclear to what extent such transfers can replace the contributions that a migrant would make to the household if s/he were present. The impact of a migrant’s absence is particularly pertinent to the context of child well-being, but relatively few empirical studies have attempted to define and measure multiple aspects of child well-being and its association with migration. Relatively little research has assessed potential trade-offs between increased material resources and the less-easily quantified consequences of parental absence such as the availability of child supervision (Kandel and Kao 2001); this is especially true of Moldova and Georgia, where limited research has explored the specific channels through which migration can affect the well-being of children. As with other Eastern European and former Soviet states, Moldova and Georgia have experienced a rapid rise in emigration that has inspired concern among policy makers and civil society organisations regarding the potential impacts these growing migration flows may have on society. While public discourse generally recognises the inflow of remittances as a positive outcome of migration, migration itself is otherwise generally regarded as deleterious for the societies and families involved in it.

This paper bridges this gap by elaborating a multidimensional well-being index for children in Moldova and Georgia, which enables the well-being outcomes of children with and without migrant parents to be compared. The index builds on the Alkire and Foster (2011) methodology for the measurement of multidimensional poverty, the method underlying the multidimensional human development index that has been published in the Human Development Report annually since 2010 by UNDP. The well-being index is constructed around six domains of wellness representing different facets of a child’s life: education, physical health, housing conditions, protection, communication access, and emotional health. This more holistic conceptualisation of child well-being enables exploration of how migration can possibly influence child well-being beyond traditional income or material well-being measures. The index has also been constructed to enable cross-country comparison of outcomes, which provides important analytical power to the method, particularly as it allows for discussion of how deviations in country context correspond to different well-being outcomes.

The next section of this paper explores the theoretical relationship between migration and well-being and provides a brief overview of previous studies on the potential effects of migration on child well-being. The third section then reviews how child well-being should be defined and operationalised. Brief backgrounds of both Moldova and Georgia are provided before the data used in the analyses are described. The indicators and methodology for constructing and using the specified child well-being index are then explained, followed by a summary of results. This paper concludes with a discussion of the results.

## Migration & Well-Being

By assessing the impacts of migration on child well-being, an implicit assumption is made that migration bears consequences for the individuals and households it affects. Migration and the well-being of children ‘left behind’ can be expected to be linked through several avenues, the most obvious of which is that migration may involve the withdrawal or addition of household-level resources that may be used to support child well-being.

Neo-classical theories of migration, such as the new economics of labour migration (NELM) theory (Stark and Bloom 1985), suggested that the migration decision is made on a household level in response to anticipated costs and benefits of migration. Within this theory, migration is expected to be mutually beneficial for both the migrant and the sending household; the household will accept some of the costs associated with migration in return for remittances, which are a means of not only expanding household income but of diversifying its sources (Massey et al. 1993; Taylor 1999; Stark and Bloom 1985). As household members, children would be expected to benefit from the resources provided by migrants, particularly if used for expenditures such as healthcare and education.

The resources a migrant can potentially share with the household in the country of origin can include not only financial capital, but can also include human capital, through the transmission of knowledge, values, and ideas in the form of “social remittances” (Levitt 1998; Acosta et al. 2007). Several studies have explored the potential uses of both financial and social remittances for children ‘left behind’. Yang (2008) in the Philippines and Mansuri (2006) in Pakistan both suggested that the receipt of remittances can loosen household economic constraints, enabling children to pursue education and reducing child labour rates. Other studies have found a positive relationship between migration and child health outcomes, as remittances can be spent on higher quality foods, vitamins, and medicines (Salah 2008) as well as in preventative and curative healthcare (Cortés 2007). Other studies in countries such as Guatemala (Moran-Taylor 2008), El Salvador de la) Garza 2010, the Philippines (Edillon 2008; Yang 2008), and Pakistan (Mansuri 2006) have found strong associations between the receipt of remittances and higher rates of educational attainment, greater rates of participation in extra-curricular activities, and better grades.

Many studies have noted that remittances are a main means through which migration can affect child well-being, but the act of migration is no guarantee that a migrant will send remittances. Particularly when migration is undertaken as a survival strategy and is funded through loans, children in migrant households may be placed in an even more tenuous economic situation than prior to migration, particularly if they shoulder the migration debt burden (van de) Glind 2010). In some situations, as a study of Kandel (2003) in Mexico found, migration may increase child labour rates, particularly among male children who must work to support the household. While remittances may enable greater expenditure on healthcare inputs, positive outcomes may develop only over time (McKenzie 2007; Hildebrandt et al. 2005). Migration can also bear negative potential consequences for child educational outcomes, with studies in Albania (Giannelli and Mangiavacchi 2010), Ecuador (Cortés 2007), and Moldova (Salah 2008) finding a relationship between parental absence and higher rates of school absenteeism, declining school performance, and declining graduation rates.

The potential impacts of migration and child well-being can seldom be neatly designated as “positive” or “negative”; the relationship between migration and child well-being outcomes is dynamic and conditional on factors such as a child’s age, post-migration caregiving arrangements, a household’s socio-economic status, and the retained ties between a migrant and the household members remaining in the origin country (e.g., Cortés 2007; Moran-Taylor 2008; Mazzucato 2014). The generalizability of insights provided from past studies is generally limited, as few studies have used data on children specifically. Among those studies that have explicitly focused on children in migrant households, few have explored the situation of children remaining in the country of origin, and fewer still have engaged an appropriate control group against which the outcomes of children in migrant households can be compared (Graham and Jordan 2011). Past studies have also largely focused on singular aspects of well-being such as physical health or educational outcomes, but given the complex interplay between migration and the conditions that affect household members, a more encompassing assessment of migration’s impact on well-being is needed. The present study builds on the insights and suggestions of past research by defining and operationalising well-being in a holistic, multidimensional well-being framework that allows comparison of wellness across dimensions as well as across two so far understudied countries.

## Defining Well-Being

One of the first challenges faced in the assessment of child well-being is in defining the concept. The components of child well-being, while shared to a certain extent with that of adults, differ according to the specific needs and vulnerabilities children face (White et al. 2003; Brooks-Gunn and Duncan 1997; Waddington 2004). In acknowledging that children are a unique population group with differentiated needs, children must be emphasised as the unit of observation.

As for any population group, decomposing the components of child well-being or poverty requires a conceptual basis. One of the most important sources for defining child deprivation—and its end result, poverty—are international instruments such as the Convention on the Rights of the Child (CRC), which provides a rights-based framework for approaching well-being. The CRC, which was adopted by the UN General Assembly in 1989, is an instrument for promotion and protection of children’s rights that outlines minimum standards for “the treatment, care, survival, development, protection and participation that are due to every individual under age 18” (UNICEF 2009; pg. 2). Within the CRC children are envisioned as rights holders, yet this entitlement to rights is both challenged and complemented by dependence on families, communities, and societies to attain minimum standards of wellness. Within this rights-based framework, child well-being can be understood as the realization of children’s rights and the fulfilment of opportunities for a child to reach his/her potential (Bradshaw et al. 2007). Interpreted this way, well-being in the context of child’s rights has strong parallels with the human development and capabilities approach championed by Amartya Sen. The capabilities approach envisions well-being as the product of an individual’s effective opportunities or capabilities to attain a desired outcome; lack of capabilities, or the freedom to choose among them, limits the range of realizable functionings, leading to deprivation or poverty (Sen 1993; Robeyns 2005). Both the child’s rights-based framework and capability approach envision well-being as inherently multidimensional, comprised of opportunities and entitlements in multiple facets of life; deprivation in single dimensions can thus lead to failure to attain well-being in total (Alkire 2002; Sen 1993; Robeyns 2005; Alkire and Foster 2011).

To translate concepts of well-being into functional measurement instruments, a list of dimensions of well-being—and the indicators by which they can be measured—must be elaborated. A significant body of literature has addressed the multidimensional nature of child poverty (see Roelen and Gassmann 2008, for a review), much of which has adopted a rights-based perspective to define well-being domains (Alkire and Roche 2011). Based on reviewed literature, functionality in a cross-cultural context, and availability of data, the following definition of child well-being is operationalized in this study:

Well-being is a multidimensional state of personal being comprised of both self-assessed (subjective) and externally-assessed (objective) positive outcomes across six realms of rights and opportunity: education, physical health, housing conditions, protection, access to communication, and emotional health.

This definition recognises the inherent complexity and multidimensionality of well-being. Individual components of well-being and their expression are the products of on-going and dynamic processes that change the risk factors and resources within a child’s immediate and more distant development environment (Bradshaw et al. 2007). Migration is one such process that alters the context in which individuals develop and function, but its effects are not universal and homogenous.

## Country Backgrounds

Moldova and Georgia both provide rich contexts in which to explore the possible relationship between parental migration and child well-being given the rapid mobility transitions both countries have experienced. Despite some commonalities in terms of the origin of large-scale emigration flows, Moldova and Georgia differ in important ways in terms of contemporary migration flows.

Following the dissolution of the Soviet Union and subsequent independence in 1991, significant emigration from Moldova began in response to sharp economic declines. The loss of the separatist territory Transnistria and the downturn of the Russian economy at the end of the 1990s contributed to the dire economic situation Moldova found itself in 1999: gross domestic product was just 34% of the level experienced a decade earlier (Pantiru et al. 2007; CIVIS/IASCI 2010), and 71% of the population lived below the poverty line (IMF 2006). The extreme level of economic vulnerability provided the first initial “push” for large-scale emigration, which has continued relatively unabated since (CIVIS/IASCI 2010). As of 2013 over 859,400 people—equivalent to 24.2% of the total population—were estimated to live abroad, the majority of whom were in the Russian Federation, Ukraine, Italy, and Romania (World Bank 2015). In 2010 most migrants were thought to be of prime working age, with approximately 80% between the ages of 18 and 44 (CIVIS/IASCI 2010).

Mobility trends in Georgia bear some similarity to those of Moldova, but the origin of large-scale migration in the post-Soviet period differs. Immediately following independence, migration flows were strongly characterised by the ethnic return of non-Georgians to countries such as Russia, Greece, and Israel as well as by conflict-induced displacement that promoted both internal and international migration (CRRC 2007). Internal conflict and ethnic strife during the early 1990s resulted in a several waves of migration, and the 2008 Russian-Georgian war over the territory of South Ossetia prompted some additional migration both within and beyond Georgia. As in Moldova, Georgia also experienced the deterioration of the economic system and state infrastructure, and despite reforms and political transitions in the early 2000s, wide-scale poverty and economic insecurity have remained a concern, with over half of the population living under the national poverty line in 2007 (Hofmann and Buckley 2011). Persistent economic insecurity contributed to continuing emigration: as of 2013, the emigrant stock represented 16.6% of the total population (World Bank 2015). The Russian Federation, Ukraine, Greece, Armenia and Uzbekistan represented the most important destination countries for migrants in 2013 (World Bank 2015).

The different origins of migration flows from Moldova and Georgia correspond to different migration experiences for individuals from each country. While the migration stream from Moldova can be considered relatively “immature”, with low rates of settlement and family reunification in destination countries (CIVIS/IASCI 2010), emigration from Georgia has included more significant levels of settlement in host countries and lower rates of return, particularly among those individuals and households that left during the conflict period (CRRC 2007). Moldovan emigration is characterized by high levels of circularity, facilitated by favourable visa regimes with the Russian Federation and by access to the European Union among dual Moldovan-Romanian passport holders. Many Georgian emigrants are in a more disadvantaged position, particularly those residing in the EU without legal right to residency or work. Such differences in settlement patterns and legal regimes may translate into different interactions between migrants abroad and their households in the origin country, making Moldova and Georgia valuable comparative cases in understanding the relationship between migration and child well-being. While comparison of these specific countries may be telling of wider trends within the former Soviet space, understanding migration and family dynamics within this region can be instructive of how migration and child well-being intersect in other countries facing similar, overlapping transitions. Given the previous literature assessing the potential impacts of migration on children remaining in the home countries, predominantly focused on the South-East Asia and Latin American regions, the assessment of how migration and child well-being are related in the post-Soviet context can provide an expanded basis for comparison of dynamics.

## Data & Methodology

The multidimensional child well-being index proposed and explored in the following analyses makes use of nationally-representative household data collected in Moldova and Georgia as part of a project that explored the potential consequences of migration for vulnerable populations “left behind”. In Moldova the household survey was implemented between September 2011 and March 2012; data was collected on 3571 households, of which 1983 contained one or more children under the age of 18. In Georgia the household survey was conducted between March and December of 2012 and captured information on 4010 households, of which 2394 contained one or more children. The survey was conducted in all regions of both countries except for the breakaway territory of Transnistria in Moldova and the de facto independent regions of Abkhazia and South Ossetia in Georgia.

Within this survey, information was collected on specific aspects of children’s lives. Caregivers of children in the household provided information about each child’s physical and emotional health, educational behaviours, and time allocation. Household-level features such as quality of housing were assumed to apply to all household members equally. This information was collected from the primary respondent in each household. Information was collected on all children in the household aged 18 or below, but the following analysis focuses on school-aged children, those aged five to 17. The rationale for excluding children younger than five is driven by the definition and comparability of well-being indicators. Very young children have different needs that require different well-being indicators. Furthermore, data on emotional well-being through the Strength and Difficulties Questionnaire was not collected for the youngest child age cohorts. The upper age limit reflects the definition of a child according to the CRC.

The survey also collected data on the migration histories of all household members, including the years of first and last migration. A migrant was defined as a person who had lived abroad for three or more months consecutively at one time. Children living in households where the migrant was not a mother or father were excluded from the analysis, as well as children with a returned migrant parent (to enable clearer comparison between children with current migrant- and non-migrant parents). Table 1 below provides an overview of characteristics of households used in the analysis.

**Table 1:** Characteristics of households containing one or more children aged 5–17

	Moldova		Georgia	
	Parent migrated	No parent migrated	Parent migrated	No parent migrated
Observations	247	552	287	543
% of households	26.0	74.0	11.0	89.0
Average HH size	4.3	4.3	4.4	4.4
Average HH dependency ratio	1.2	1.0	1.1	1.1
Average n° people employed in the HH	0.5	1.2	0.4	0.8

Authors’ calculation. Sample is weighted to represent total population. Note: dependency ratio is the ratio of children and elderly in the household to the number of working-age adults; all results represent sample averages unless indicated otherwise

Descriptively, the two survey samples differ from one another. The sample collected in Moldova was slightly larger than that collected in Georgia. A greater number of migrant households were sampled in Georgia, but when population weights are applied to accommodate oversampling, the share of parent-away households in the Georgian sample is significantly smaller than the sample of such households in Moldova. The features of migrants included in the analytical sample also differed between the two countries, which can be seen in Table 2.

**Table 2:** Demographic characteristics of migrants, weighted for total population

	Moldova	Georgia
Sex		
Male	67.4%	49%
Female	32.6%	51%
Average age	36	38
Most prevalent level of education	Upper secondary	Incomplete tertiary
% Holding a residence permit	48.5%	53.3%
% HH receiving remittances	40.6%	77.5%

Authors’ calculation

In Moldova over 67% of migrant parents were male, while in Georgia a larger proportion of migrant parents were female (51%). Georgian migrants also tended to be slightly older and to have a slightly higher level of education: while the average migrant in Moldova had attained upper secondary education, an average Georgian migrant had a secondary degree and had incomplete tertiary education. A larger proportion of households in Georgia than in Moldova received remittances, which likely reflects differences in migration patterns such as degree of circularity and duration of migration. These initial descriptive differences may suggest that the experiences of children with migrant family members differs between the two countries.

### Indicators

To analyse and compare the rates of multidimensional well-being between children with and without migrant family members, a child-specific well-being index was constructed with six dimensions of child well-being: education, physical health, housing conditions, protection, communication access, and emotional health. The current analysis drew from measurement tools expressly designed for the particular population of interest (children aged 5–17). Both Moldova and Georgia adopted the Convention on the Rights of the Child (CRC), which provides some guidance to the selection of indicators related to fundamental well-being standards that every child has the right to enjoy. The choice of indicators also allows for comparison of child well-being between the two countries. Table 3 contains the list of dimensions and indicators chosen for the measurement of child well-being.

**Table 3:** Well-being indicators per dimension

Education	
Child attends school at the appropriate grade	
Physical health	
Child has received all vaccinations	
Housing well-being	
Child is living in a household with appropriate flooring, water, and electricity	
Communication	
Child lives in a household with a mobile phone	
Protection	
Child is not physically abused	
Emotional well-being	
Child attains a normal score on the strengths & difficulties questionnaire	

The educational well-being dimension is measured by school enrolment; for children aged five and six, school enrolment is measured by pre-school attendance, and for children aged seven and older, this indicator measures enrolment in the appropriate grade for a child’s age. Physical health is measured by a child’s receipt of the full regime of required vaccinations, which includes BCG, DPT, measles, and hepatitis B. Housing conditions are measured by access to electricity, proper flooring (e.g., not dirt or concrete), and a safe source of drinking water (e.g., not surface water or water from tenuous sources like rainwater collection). The dimension of protection is measured by whether a caregiver reports repeatedly beating a child as punishment. Communication well-being is measured by access to a modern source of communication, in this case a mobile phone. While this indicator is measured on the household level, it can be expected that children living in households with technologies that facilitate communication will benefit individually from the greater level of connectedness. Finally, emotional well-being is measured by the total difficulties score of the Strength and Difficulties Questionnaire (SDQ), a behavioural screening instrument that uses 25 questions on psychological attributes to identify potential cases of mental health disorder (Goodman 1997). In contrast to other child well-being indices that include indicators of material well-being such as household income or expenditure, the index proposed here consciously omitted such indicators because they are likely to influence the attainment of well-being across all dimensions. Household poverty status, measured as having an adult equivalent expenditure below 60% of the median, is included as a control variable in all subsequent analyses. The indicators included in this index were chosen because they were both relevant and available in both countries, which enables comparison of the same concepts across differing contexts. They were also chosen for their ease of interpretation, as each indicator has a clear threshold for when a child does and does not meet acceptable levels of well-being.

### Methodology

Child well-being was calculated in two steps. A child is considered not deprived if s/he meets the established well-being threshold set for a given indicator. Indicator well-being rates *(IWB)* are calculated by counting the number of children who passed the defined threshold, expressed as a share of all children (Roelen et al. 2011):

$$ { I WB}_x=\frac{1}{n}\sum_{i=1}^n{I}_{i x} $$

where *n* is the number of children for which the indicator is observable and *I*_*ix*_ is a binary variable taking the value 1 if child *i* has reached the threshold and 0 if the child has not with respect to indicator *x*.

A second step involved building a multidimensional well-being index inspired by the Alkire and Foster (2011) methodology for the measurement of multidimensional poverty. A child is considered to be multidimensionally well if the weighted combination of dimensions is equal to or exceeds 70% of the total; in this index, a child must be well in at least four of six indicators to be considered multidimensionally well. Each domain is assigned equal weight, which facilitates the interpretation of results (Atkinson 2003) but also asserts that each dimension is considered of equal importance. The decision to set the cut-off at 70% follows the cut-off used for multidimensional child well-being indices (Roelen and Gassmann 2014).

Children with and without migrant parents can then be compared. Multivariate analysis is applied to control for and identify other correlates that determine child well-being, such as personal characteristics of the child and regional or household characteristics. Separate binary outcome models are estimated for selected indicators using standard probit models, specified as:

$$ \Pr \left({y}_i=1\left|{x}_i\right.\right)=\Phi \left({x}_i\beta \right),\mathrm{with}\ \mathrm{i}=1,\dots, \mathrm{N} $$

Where *y*_*i*_ is the binary outcome variable, *Φ* is the standard normal distribution function, ***x***_***i***_ is a vector of explanatory variables, and ***β*** is a vector of coefficients to be estimated. In the following analysis, the dependent variable is the probability that an individual is well with respect to a specific indicator. In order to assess whether the effect of migration is significantly different between countries, models for each country are estimated separately, and a Wald chi square test is performed to establish if the coefficients for migration status significantly differ from each other. The formula for this statistic is written as:

$$ \frac{{\left({b}_M-{b}_G\right)}^2}{{\left[ se\ \left({b}_M\right)\right]}^2+{\left[ se\ \left({b}_G\right)\right]}^2} $$

where *b*_*M*_ is the coefficient for Moldova and *b*_*G*_ is the coefficient for Georgia.^2^ Differences in the migration coefficients may not necessarily indicate true differences in causal effects, for example when the two models differ in the degree of residual variation (or unobserved heterogeneity). If this is the case, the test would report a misleading result, as the differences in the migration coefficient would be driven by other unobserved correlates that are not included in the model. To correct for potential deviation in residual variation, ordinal generalized linear models are used that estimate heterogeneous choice models that allow for heteroskedasticity for the specified variables (in this case, the country).^3^

The following section describes the results of the multidimensional index. Descriptive statistics for indicator- and multidimensional well-being are presented first in which group differences both within and between countries are tested. Results of these bivariate analyses are followed by the results of the multivariate analyses, which assess the relationship between migration and child well-being outcomes when accounting for other factors that can help predict child well-being.

## Results

Table 4 provides an overview of well-being rates achieved by children in each study country for each indicator and on the total multidimensional well-being index. In Moldova, achieved rates of well-being ranged from 74% in the domain of housing well-being to 96% within the protection domain. With respect to the combined well-being index, 84% of children can be considered well, which reflects the overall high level of child well-being across the six dimensions. Children in Georgia expressed a similar level of overall well-being, with over 83% considered well on the total index level. Children in Georgia achieved the worst outcomes in the domain of physical health, with only 73.5% considered well, and the best outcomes in the domains of protection and emotional well-being, where 94% were considered well.

**Table 4:** Domain and multidimensional well-being rates

Moldova		Education	Health	Housing	Protection	Communication	Emotional	MWI
	*n*	%	%	%	%	%	%	%
Migrant	370	92.5	87.0	72.6	97.3	87.0	93.7	84.8
Father abroad	165	91.8	85.0	70.3	97.4	89.5	92.8	83.6
Mother abroad	134	93.0	86.4	72.9	95.8	86.7	95.3	87.2
Both parent abroad	71	93.4	89.6	78.2	100	80.7	92.8	83.5
Non migrant	964	91.8	89.6	75.6	95.4	85.9	93.4	83.9
Total	1334	92.0	88.8	74.2	95.8	86.1	93.4	84.1
Significance								
Georgia								
	N	%	%	%	%	%	%	%
Migrant	421	95.5	78.5	83.2	97.1	97.3	92.8	90.9
Father abroad	206	95.1	77.2	85.5	94.8	97.8	88.7	90.7
Mother abroad	181	95.2	82.1	83.9	99.7	96.5	96.6	92.6
Both parents abroad	34	98.9	70.6	68.6	97.9	97.6	97.9	84.8
Non migrant	882	92.9	72.9	76.5	93.9	92.3	94.5	82.1
Total	1303	93.2	73.5	77.2	94.3	92.8	94.4	83.0
Significance					**	**	**	**

Authors’ calculations. Note: *** p < 0.01; ** p < 0.05; * p < 0.1 significance levels based on chi2 test of independence comparing father abroad, mother abroad, both parents abroad and no migrant

Children in Moldova in migrant and non-migrant households did not attain statistically-different outcomes in any domain. In Georgia, children who had a parent abroad were better off in the dimensions of protection and communication and in the overall multidimensional index than children without migrant parents. Children with migrant parents were significantly worse off than children in non-migrant household in the dimensions of emotional well-being, however. Such differences appear to be associated with the absence of particular individuals: a father’s migration was associated with worse outcomes in the protection and emotional well-being domains, whereas more limited differences in outcomes were associated with a mother’s migration or the migration of both parents. The absence of both parents corresponded to worse outcomes for overall well-being measured by the combined index. Based on the bivariate analysis, migration appears to be a more important factor in shaping the well-being outcomes of children in Georgia than in Moldova.

To determine the extent to which the migration of a parent corresponds to differences in child well-being outcomes when accounting for other relevant covariates, multivariate analyses utilising probit models were also conducted. The results are summarised in Table 5, which indicates the marginal effects of migration status for each aspect of well-being.^4^

**Table 5:** Marginal effect of migration status as a determinant of well-being

	Model		
Dimension	Moldova	Georgia	Test^a^
Education	0.02 (0.02)	-0.02 (0.03)	
Health	-0.03 (0.03)	0.07 (0.06)	
Housing	0.01 (0.03)	0.12^*^(0.05)	*
Communication	0.02 (0.03)	0.10^*^ (0.05)	
Emotional	-0.02 (0.02)	-0.02 (0.02)	
Protection	0.05^*^ (0.02)	0.00 (0.03)	
MWI	0.02 (0.03)	0.14^**^ (0.05)	+
N° Observations	1334	1303	

Authors’ calculations. Reported results are average marginal effects (dx/dy) for children living in migrant households. Other control variables not reported. Robust standard errors in parentheses; +p < 0.1; * p < 0.05; ** p < 0.01. ^a^Differences between countries in the migration coefficient are significant at a + 10% level, *5% level, and **1% level based on Wald chi square test (corrected for unequal residual variation or unobserved heterogeneity)

The multivariate analysis confirms some of the results of the bivariate analysis, namely that migration appears to have a more significant effect on the well-being of children in Georgia than in Moldova. Children in migrant households in Georgia had higher probabilities of being well in the domains of housing, communication, and on total index level than children in non-migrant households. In Moldova, migration was significantly associated with higher probabilities of being considered well in the protection domain but was otherwise non-significant. Significant differences between countries can be observed in the housing dimension and with respect to the combined multidimensional well-being index; in both cases, migration in Georgia was positively correlated with well-being, whereas in Moldova the migration coefficients were not significant.

The extent to which parental migration is related to child well-being not only depends on whether there is a migrant parent in the household but also on who migrates and who adopts the role of the caregiver in the household. Tables 6 and 7 show the results of the extended models. The absence of either the mother or the father is positively correlated with well-being in the dimensions of housing and access to communication in Georgia, as well as on the overall child well-being. The positive correlation between parental migration and well-being in the communication dimension may be the direct effect of the desire of parents to stay in regular contact with their children. For Moldova the absence of the father has a positive effect on well-being in the protection domain, indicating that these children less frequently experience physical abuse.

**Table 6:** Marginal effects of migration status as a determinant of well-being: Georgia

	Education	Health	Housing	Communication	Emotional	Protection	MWI
Migration status (ref category: no migration)							
Mother migrated	-0.07	0.10	0.21^**^	0.08^+^	0.03	0.04	0.18^**^
	(0.05)	(0.08)	(0.07)	(0.04)	(0.03)	(0.04)	(0.06)
Father migrated	-0.00	0.06	0.10^+^	0.10^+^	-0.04	-0.00	0.11^*^
	(0.03)	(0.07)	(0.05)	(0.06)	(0.02)	(0.03)	(0.06)
Both parents migrated	-0.05	0.02	0.08	0.12^*^	0.05	-0.05	0.10
	(0.06)	(0.14)	(0.14)	(0.06)	(0.05)	(0.07)	(0.11)
Male	-0.02	-0.02	-0.03	-0.00	-0.04^**^	-0.01	-0.00
	(0.01)	(0.03)	(0.03)	(0.02)	(0.01)	(0.01)	(0.02)
Caregiver (ref category: mother)							
Father	0.02	-0.10	-0.04	0.05	-0.03	-0.01	-0.09
	(0.06)	(0.07)	(0.07)	(0.04)	(0.03)	(0.04)	(0.06)
Grandparent	0.06^+^	-0.01	-0.18^**^	-0.03	-0.03	0.09^**^	-0.10^+^
	(0.03)	(0.07)	(0.06)	(0.03)	(0.03)	(0.03)	(0.05)
Other relative	0.09^*^	-0.20^*^	0.00	-0.09^*^	-0.00	0.01	-0.16^*^
	(0.04)	(0.08)	(0.09)	(0.04)	(0.04)	(0.05)	(0.07)
Age	0.12^**^	0.03	0.00	-0.03^+^	-0.02	-0.01	0.05^*^
	(0.01)	(0.03)	(0.02)	(0.02)	(0.01)	(0.02)	(0.02)
Age squared	-0.01^**^	-0.00	-0.00	0.00^*^	0.00	0.00	-0.00^*^
	(0.00)	(0.00)	(0.00)	(0.00)	(0.00)	(0.00)	(0.00)
Urban	-0.00	-0.12^**^	0.22^**^	0.06^**^	-0.00	-0.02	0.07^*^
	(0.02)	(0.03)	(0.03)	(0.02)	(0.02)	(0.01)	(0.03)
Highest achieved education (ref category: Higher)							
Lower/upper secondary	-0.05	-0.39^**^	-0.08	-0.12^**^	-0.13^**^	-0.07^+^	-0.30^**^
	(0.04)	(0.09)	(0.08)	(0.04)	(0.04)	(0.04)	(0.07)
Post-secondary	-0.05^**^	0.02	-0.04	-0.05^**^	0.01	-0.03^+^	-0.09^**^
	(0.02)	(0.04)	(0.03)	(0.02)	(0.02)	(0.02)	(0.03)
Number of siblings	0.01	0.03	-0.05^*^	-0.00	0.00	0.01	0.01
	(0.01)	(0.02)	(0.02)	(0.01)	(0.01)	(0.01)	(0.02)
Number of adults	0.00	0.02^+^	-0.01	0.01^+^	0.01	0.00	0.01
	(0.01)	(0.01)	(0.01)	(0.01)	(0.01)	(0.01)	(0.01)
poverty status	0.03	-0.02	-0.02	-0.06^**^	-0.03^+^	0.00	-0.06^*^
	(0.03)	(0.04)	(0.03)	(0.02)	(0.02)	(0.02)	(0.03)
HH received remittances	0.01	0.06	-0.01	-0.05	0.02	0.02	0.03
	(0.04)	(0.06)	(0.05)	(0.04)	(0.02)	(0.03)	(0.05)
Observations	1303	1303	1303	1303	1303	1303	1303
F stat	5.7	4.1	7.7	6.0	2.7	3.3	5.2
Prob > F	0.00	0.00	0.00	0.00	0.00	0.00	0.00

Source: Authors’ calculations. Reported results are average marginal effects (dx/dy) for children living in migrant households

Robust standard errors in parentheses; +p < 0.1; * p < 0.05; ** p < 0.01

**Table 7:** Marginal effects of migration status as a determinant of well-being: Moldova

	Education	Health	Housing	Communication	Emotional	Protection	MWI
Migration status (ref category: no migration)							
Mother migrated	0.04	-0.02	0.04	0.02	0.02	0.02	0.09^+^
	(0.04)	(0.05)	(0.06)	(0.04)	(0.03)	(0.03)	(0.05)
Father migrated	0.01	-0.04	-0.01	0.02	-0.03	0.06^**^	-0.01
	(0.02)	(0.03)	(0.04)	(0.03)	(0.03)	(0.02)	(0.03)
Both parents migrated	0.06	0.01	0.08	0.02	-0.00	0.03	0.05
	(0.04)	(0.06)	(0.08)	(0.05)	(0.04)	(0.04	(0.06)
Male	-0.01	-0.03	0.01	0.00	-0.03^+^	-0.03^*^	-0.01
	(0.01)	(0.02)	(0.02)	(0.02)	(0.01)	(0.01)	(0.02)
Caregiver (mother)							
Father	-0.06^+^	-0.03	-0.07	0.05	-0.04	-0.00	-0.06^+^
	(0.03)	(0.04)	(0.05)	(0.03)	(0.03)	(0.03)	(0.04)
Grandparent	-0.03	-0.03	0.02	-0.01	-0.01	0.05^+^	0.01
	(0.03)	(0.04)	(0.05)	(0.03)	(0.03)	(0.03)	(0.04)
Other relative	-0.02	-0.14^+^	0.23^*^	-0.11^+^	-0.01	0.02	-0.14^+^
	(0.06)	(0.08)	(0.10)	(0.07)	(0.06)	(0.04)	(0.08)
Age	0.09^**^	0.06^**^	0.03	0.01	0.00	-0.02^*^	0.07^**^
	(0.01)	(0.02)	(0.02)	(0.01)	(0.01)	(0.01)	(0.02)
Age squared	-0.00^**^	-0.00^**^	-0.00	-0.00	-0.00	0.00^*^	-0.00^**^
	(0.00)	(0.00)	(0.00)	(0.00)	(0.00)	(0.00)	(0.00)
Urban	0.01	-0.08^**^	0.33^**^	0.19^**^	0.00	0.04^*^	0.14^**^
	(0.02)	(0.03)	(0.04)	(0.04)	(0.02)	(0.02)	(0.04)
Highest achieved education (ref category: Higher)							
Lower secondary	-0.04	-0.04	-0.13^**^	-0.18^**^	-0.02	-0.03^*^	-0.18^**^
	(0.02)	(0.03)	(0.04)	(0.03)	(0.02)	(0.01)	(0.03)
Upper secondary	-0.01	-0.02	-0.02	-0.13^**^	-0.03	0.04^+^	-0.08^*^
	(0.02)	(0.04)	(0.04)	(0.03)	(0.02)	(0.02)	(0.03)
Post-secondary	-0.01	-0.02	-0.02	-0.13^**^	0.01	0.02	-0.05
	(0.02)	(0.03)	(0.03)	(0.03)	(0.02)	(0.02)	(0.03)
Number of siblings	-0.02^*^	-0.00	-0.03^*^	-0.01	-0.01	-0.01^+^	-0.02^*^
	(0.01)	(0.01)	(0.01)	(0.01)	(0.01)	(0.01)	(0.01)
Number of adults	-0.01^*^	0.00	-0.02^+^	0.02^*^	-0.01	0.00	-0.02^+^
	(0.01)	(0.01)	(0.01)	(0.01)	(0.01)	(0.00)	(0.01)
Poor	-0.01	0.00	-0.04	-0.20^**^	-0.02	-0.01	-0.12^**^
	(0.02)	(0.03)	(0.03)	(0.02)	(0.02)	(0.01)	(0.02)
HH received remittances	-0.00	0.00	-0.01	0.02	0.05^+^	0.06^**^	0.01
	(0.03)	(0.04)	(0.04)	(0.03)	(0.03)	(0.02)	(0.03)
Observations	1334	1334	1334	1334	1334	1334	1334
F stat	6.5	2.2	6.6	12.7	1.2	6.4	11.0
Prob > F	0.00	0.00	0.00	0.00	0.26	0.00	0.00

Authors’ calculations. Reported results are average marginal effects (dx/dy) for children living in migrant households

Robust standard errors in parentheses; +p < 0.1; * p < 0.05; ** p < 0.01

The specific caregiver of a child is significant for some dimensions, and can be either positive or negative. In Georgia, having a grandparent as primary caregiver (as compared to the mother) increases the likelihood of well-being in education and protection but decreases the likelihood of attaining housing and overall multidimensional well-being. If a non-parent relative is the caregiver the likelihood of being well-off is lower in the health and communication dimension, as well as in the multidimensional well-being index. Similar effects can be observed in Moldova. Children with a non-relative as main caregiver fare worse in the domains of health, communication, and the overall well-being index but they have higher probabilities of being well in the housing domain compared to children cared for by a mother. Having a grandparent caregiver is positively associated with protection well-being, while a father as caregiver seems to negatively affect the likelihood of attending school at the appropriate grade.

Beyond parental migration status and type of caregiver, variables like household composition, household educational attainment, location, and child age are important determinants of child well-being in both Moldova and Georgia. The likelihood of education well-being increases with age in both countries, although the relationship may not be linear. Higher educational attainment in the household is positively and significantly associated with most well-being dimensions in both countries. Living in an urban environment is negatively correlated with health well-being but is positively correlated with well-being in housing, access to communication, and protection. In Moldova female children have a higher probability than male children of being well in the protection domain, and girls in both countries have higher probabilities of achieving emotional well-being than boys. The number of siblings is also important in Moldova for determining well-being, as a higher number of co-resident children corresponds to decreased chances of attaining housing, protection, and educational well-being. In Georgia, this variable is correlated (negatively) to housing well-being.

## Discussion

In contrast to past studies that have found strong ties between parental absence through migration and differences in child well-being outcomes, these analyses of the relationship between parental migration and multidimensional child well-being suggests limited differences in the well-being outcomes of children with and without migrant parents.

Three observations arise from these results: 1) in both Moldova and Georgia, rates of child well-being are generally high, but important differences in well-being attainment can be observed across dimensions; 2) the relationship between parental migration and child well-being not only varies by who specifically has migrated but also by the domain of well-being measured, and; 3) the influence of parental migration on child well-being varies by country. Each of these observations calls for greater reflection of how child well-being is shaped by larger societal and family processes. While one of these processes is migration, it is important to recognise that migration may bear a more limited influence on child well-being than other factors, and migration may itself be a proxy or manifestation of other processes (such as economic transition) that affect child well-being through other channels.

Results highlight that well-being can (and should) be explored through its constituent parts, as children who may appear well on an aggregate level may nevertheless experience low levels of wellness in specific areas. The difference in rates of well-being attainment across dimensions also highlights the role of specific factors in contributing to well-being. For example, in both countries the physical location of a household (i.e., in an urban or rural area) corresponded to significant differences in outcomes across several domains of wellness, with children in rural areas expressing lower probabilities of being well in housing and communication in both countries.

Parental migration corresponded to meaningful differences in specific aspects of well-being inconsistently, as only particular forms of parental migration related to differences in well-being outcomes. Results signal that parental migration seldom bears a significant influence on the attainment of well-being, but when it does, that relationship is generally positive and low in magnitude. Such results may reflect household-level coping and coordination mechanisms. Who specifically has migrated may imply who acts as a child’s primary caregiver, and it is likely a combination of these traits that explains differing patterns of well-being attainment across dimensions. For example, in mother-away households, another family member—most often a grandmother—may assume responsibility for daily child-care activities. In Georgia, the caregiving transition is likely to be smooth, as grandparents often play an intense role in child-rearing even before migration, and parents who do engage in migration are likely to be enabled to do so given support by members of the extended family (Hofmann and Buckley 2011).

Finally, the relationship between different forms of parental migration and child well-being differed widely between the study countries. Migration corresponded to limited differences in child well-being in Moldova yet corresponded to greater probabilities of children being considered well in several domains in Georgia. Differences between the countries may reflect differences in migration trajectories and the processes by which individuals are “selected” into migration in the first place. Greater shares of Moldovan migrants than Georgian migrants were destined for countries in the Commonwealth of Independent States, namely the Russian Federation, where many migrants work in insecure and volatile sectors such as construction and agriculture. Given the souring relationship between Georgia and Russia, recent migration flows have turned more towards countries in and beyond the European Union, where many migrants work in home-, child-, and elder-care functions. Differences in the industries in which Moldovan and Georgian migrants work may correspond to differences in job security and exposure to unemployment or wage withholding, which may carry over into the costs and benefits the origin-country household bears for the migration of an individual member. Another difference between the countries is in who enters migration. Comparisons between the sample of migrants collected in Moldova and Georgia suggest that Georgian migrants are slightly better educated and older than their Moldovan counterparts. This may suggest that Georgian households that produce migrants are better off socio-economically than Moldovan households even before migration. Furthermore, Georgian migrants who are older may be less likely to have very young children, and again the predominance of multi-generational, extended households may ease caregiving transitions post-migration.

The limited yet generally positive relationship between different forms of parental migration and multidimensional child well-being in Moldova and Georgia suggests that public discourses about migration—which largely anticipate strong, negative relationships between parental absence and child well-being—may be misplaced. Particularly in Moldova, where a great deal of research has focused on the dire consequences of migration for the “left behind” (Pantiru et al. 2007), there is limited evidence to suggest that children with migrant parents suffer from that absence. The results do not dismiss the possibility that parental absence through migration can erode child well-being, but it does emphasise the need to understand how migration, family systems, and societal processes intersect to bolster or undermine child well-being and its various expressions and domains.

The need to understand child well-being (and well-becoming) in context can provide essential guidance to future research and policy designs. By decomposing child well-being into different components, and by observing how migration intersects with other resources that can feed into those components, policies aimed at enhancing or protecting child well-being may be able to better target interventions to domains of more or less acute deprivation. Rather than treating parental migration as a key distinguishing factor that *determines* deprivation, it is important to map how parental migration is accommodated in family life and to understand the common factors that influence both initial migration decisions and the opportunities a child has to achieve functional wellness in different domains.

The multiple and overlapping interactions among migration and other family- and societal processes suggests that context is essential in predicting how parental absence through migration may affect child well-being.

## Footnotes

1 In 2013, the stock of emigrants accounted for 24.2% of the Moldovan and 16.6% of the Georgian population (World Bank 2015).

2 From Allison (1999).

3 For more information on these tests, see Williams (2009) and Allison (1999).

4 Given the focus of the analysis on the role of migration, the marginal effects and significance levels of other covariates are not displayed here but are available upon request from the authors.
